# Over Eating

**Published:** 1891-05

**Authors:** 


### OVER EATING.

The habit of over eating is far too common, even with those persons who practice moderation in other ways. The day laborer may habitually indulge in an amount of food without injury, which would seriously affect a person of a less active mode of life, because his heavy work burns off the excess of food ; but in most cases the excess of food is not carried off by a so-called bilious attack, and then, if there is no work to burn up the supply, what happens ? In some constitutions dyspepsia, in others an ever-increasing bulk ; now this bulk disinclines to exertion, so that with increase of bulk less work is done, while there is a growing disinclination to exertion—even a repugnance in extreme cases to any form of exercise. These cases are among the most difficult the physician can treat, for the sufferer, though he may wish for relief, lacks the energy to find it. As a rule, stoutness is connected with errors of diet—errors of excess, perhaps oftener than people are prepared to admit, but often to errors of kind.

